# Crowdsourcing as a Novel Technique for Retinal Fundus Photography Classification: Analysis of Images in the EPIC Norfolk Cohort on Behalf of the UKBiobank Eye and Vision Consortium

**DOI:** 10.1371/journal.pone.0071154

**Published:** 2013-08-21

**Authors:** Danny Mitry, Tunde Peto, Shabina Hayat, James E. Morgan, Kay-Tee Khaw, Paul J. Foster

**Affiliations:** 1 National Institute for Health Research Biomedical Research Centre at Moorfields Eye Hospital & University College London Institute of Ophthalmology, London, United Kingdom; 2 Department of Public Health and Primary Care, University of Cambridge Strangeways Research Laboratory, Worts Causeway, Cambridge, United Kingdom; 3 School of Optometry and Vision Sciences, Cardiff University, Cardiff, United Kingdom; 4 Department of Clinical Gerontology, Addenbrookes Hospital, University of Cambridge, Cambridge, United Kingdom; The University of Queensland, Australia

## Abstract

**Aim:**

Crowdsourcing is the process of outsourcing numerous tasks to many untrained individuals. Our aim was to assess the performance and repeatability of crowdsourcing for the classification of retinal fundus photography.

**Methods:**

One hundred retinal fundus photograph images with pre-determined disease criteria were selected by experts from a large cohort study. After reading brief instructions and an example classification, we requested that knowledge workers (KWs) from a crowdsourcing platform classified each image as normal or abnormal with grades of severity. Each image was classified 20 times by different KWs. Four study designs were examined to assess the effect of varying incentive and KW experience in classification accuracy. All study designs were conducted twice to examine repeatability. Performance was assessed by comparing the sensitivity, specificity and area under the receiver operating characteristic curve (AUC).

**Results:**

Without restriction on eligible participants, two thousand classifications of 100 images were received in under 24 hours at minimal cost. In trial 1 all study designs had an AUC (95%CI) of 0.701(0.680–0.721) or greater for classification of normal/abnormal. In trial 1, the highest AUC (95%CI) for normal/abnormal classification was 0.757 (0.738–0.776) for KWs with moderate experience. Comparable results were observed in trial 2. In trial 1, between 64–86% of any abnormal image was correctly classified by over half of all KWs. In trial 2, this ranged between 74–97%. Sensitivity was ≥96% for normal versus severely abnormal detections across all trials. Sensitivity for normal versus mildly abnormal varied between 61–79% across trials.

**Conclusions:**

With minimal training, crowdsourcing represents an accurate, rapid and cost-effective method of retinal image analysis which demonstrates good repeatability. Larger studies with more comprehensive participant training are needed to explore the utility of this compelling technique in large scale medical image analysis.

## Introduction

Crowdsourcing is an emerging concept that has attracted significant attention in recent years as a strategy for solving computationally expensive and difficult problems. Crowdsourcing is the process of outsourcing numerous tasks to many untrained individuals. It is in widespread use in marketing and can deliver a productivity on a scale that is otherwise very difficult to achieve. Scientifically crowdsourcing has been popularised through its success in the categorization of galaxies. [1] In the biological sciences it has shown great potential in the determination of protein folding structure which has limited feasibility with conventional computational approaches. [2] In healthcare, crowdsourcing has been used in drug discovery, analysis of imaging, clinical diagnosis and to improve service efficiency [3]–[6].

In general there is a lot of detail and subtlety associated with the analysis of medical images. Image categorisation can, therefore be tedious and time consuming, even for highly trained professionals. One of the principal advantages of crowdsourcing in medical image analysis is the potential for a marked reduction in analysis time with attendant reductions in analysis costs. These observations are predicated on the assumption that humans are better and more flexible than machines at certain tasks.

The largest commercial crowdsourcing provider is Amazon’s Mechanical Turk. (https://www.mturk.com/mturk/welcome) MTurk is an Internet-based platform that allows requesters to distribute small computer-based tasks to a large number of untrained workers. Typically the tasks require simple categorization based on discrete and small datasets and/or images using multiple choice question format.

The large scale acquisition of retinal images has become routine in the management of disease such as diabetic retinopathy, macular degeneration and glaucoma. These datasets present a formidable challenge in terms of analysis, for which a crowdsourced approach may be feasible. We therefore evaluated the potential for crowdsourcing (also known as distributed human intelligence) as an effective and accurate method of fundus photography classification.

## Methods

The EPIC-Norfolk 3HC was reviewed and approved by the East Norfolk and Waverney NHS Research Governance Committee (2005EC07L) and the Norfolk Research Ethics Committee (05/Q0101/191). Local research and development approval was obtained through Moorfield’s Eye Hospital, London (FOSP1018S). The research was conducted in accordance with the principles of the Declaration of Helsinki. All participants gave written, informed consent.

EPIC (European Prospective Investigation of Cancer) is a pan-European study that started in 1989 with the primary aim of investigating the relationship between diet and cancer risk.[16] EPIC-Norfolk is one of the U.K. arms of the European cohort study. The aims of the EPIC-Norfolk cohort were subsequently broadened to include additional endpoints and exposures such as lifestyle and other environmental factors. The EPIC-Norfolk cohort was recruited in 1993–1997 and comprised 25,639 predominantly white European participants aged 40–79 years. The third health examination (3HC) was carried out between 2006 and 2011 with the objective of investigating various physical, cognitive and ocular characteristics of 8,623 participants then aged 48–91 years. A detailed eye examination including mydriatic fundus photography was attempted on all participants in the 3HC using a Topcon TRC NW6S camera. [7] A single image of the macular region and optic disc (field 2 of the modified Airlie House classification) was taken of each eye. [8].

A panel of two expert clinicians (D.M., P.F.) and two senior retinal photography graders selected, by consensus, a series of 100 retinal images from the EPIC Norfolk 3HC. We selected 10 severely abnormal images, 60 mildly abnormal images and 30 normal images, with pre-determined criteria to assess the discriminating efficacy of the proposed technique. Severely abnormal images were determined as having grossly abnormal findings, including significant haemorrhage, pigmentation or fibrosis. Mildly abnormal images were designated if there was a subtle abnormality such as dot haemorrhages or fine pigmentary changes. Normal images had no discernible pathology. Figures S2, S3, S4 demonstrate example images for each category. All images were anonymysed and uploaded onto an ftp site for the study duration to allow remote access.

We used the MTurk Web platform for anonymous workers to perform a classification task of the fundus photographs in our dataset. MTurk employs knowledge workers (KWs), who are untrained individuals to carry out simple tasks. KWs are registered Amazon users who have a record of completing these types of tasks. At the time of this study there were over 200,000 registered KWs. Each KW receives a small monetary reward from the requester for each task that they complete that is of a suitable standard to the requester. Amazon keeps a record of the performance of each KW and if desired, filters can be set by the requester, for example, permitting only KWs with a high success rate to perform the task. Each retinal image classification was published as one human intelligence task (HIT). For each HIT, KWs were given some background information about the nature of the photograph and a written description of abnormal features of interest. In addition, they were shown two labelled example images of normal fundus photographs as part of a basic training exercise to help distinguish normal from abnormal. KWs were asked if the test image differed from the normal image. Specifically, they were asked to determine if there were any additional features in the test image that were absent in the normal image. If the answer was ‘yes’ they were then asked to describe the nature of the additional features through a simple drop-down menu. (see Figure S1 for sample questionnaire) Each KW could only complete the same image once but there were no restrictions on the number of assignments that a KW could complete. No demographic data was collected on KWs completing the task and no nationality restrictions were placed. Based on previous estimations of repeated task accuracy in distributed human intelligence tasks, we requested 20 KW classifications per image. [4] In order to assess the effect of skill and compensation on classification accuracy we conducted four different study designs:

1. No previous experience required - compensation 0.03 cents (USD) per HIT
2. No previous experience required - compensation 0.05 cents per HIT
3. Completed ≥500 HITs with ≥90% approval - compensation 0.03 cents per HIT
4. Completed ≥5000 HITs with ≥99% approval - compensation 0.03 cents per HIT

All four study designs were repeated to determine if the findings from trial 1 were reproducible. Using the selection of images as a pre-defined reference standard, we calculated the sensitivity and specificity for each of the study designs by degree of abnormality. Receiver operating characteristic (ROC) curves were analysed. The area under the ROC plot measures discrimination and is the most commonly used global index of diagnostic accuracy. The area under the ROC curves (AUC) were calculated as non parametric Mann-Whitney estimates and comparison between curves was performed using the z statistic for correlation. As a secondary analysis, we compared the characteristics for easy classifications (distinguishing normal and severely abnormal) and difficult classifications (distinguishing normal and mildly abnormal). Where relevant, statistics are reported with associated 95% confidence intervals. All analyses were performed using STATA v12.

## Results

For each study design in trial 1 and 2, we received all 2,000 requested classifications of the 100 images selected. Table 1 illustrates the baseline characteristics for the KW participation in each of the four study designs in trial 1 and 2 highlighting a decrease in the number of KWs performing our task and a longer time to overall completion when experience eligibility restrictions were applied. The sensitivity and AUC for each study design in trials 1 and 2 by classification difficulty is shown in table 2. In trial 1, all study designs demonstrated a sensitivity of ≥98% for the correct classification of normal versus severely abnormal retinal images, which is comparable to the value of ≥96% in trial 2.

**Table 1:** Baseline characteristics of KW participation by study design for trials 1 and 2.

	Trial 1			
	0.03c	0.05c	0.03c_500_90%	0.03c_5000_99%
Number of different KWs	152	127	39	61
Mean (SD) number of HITs per KWs	13(18)	15(20)	51(96)	26(16)
Mean (SD) time on each HIT (secs)	78(109)	62(76)	63(71)	66(90)
Time to overall completion	<1 day	<1 day	1–2 days	15 days
	**Trial 2**			
	0.03_20	0.05_20	0.03_500_90%	0.03_5000_99
Number of different workers	69	72	56	46
Mean (SD) number of hits per KWs	37(18)	35(19)	25(15)	24(14)
Mean (SD) time on each hit (secs)	63(83)	73(105)	79(102)	58(80)
Time to overall completion	<1 day	<1 day	2–3 days	7 days

(0.03c = study design 1; 0.05c = study design 2; 0.03c_500_90% = study design 3; 0.03c_5000_99% = study design 4).

**Table 2:** The proportion correctly identified by severity of abnormality as well as the sensitivity, specificity and area under the ROC curve (AUC) for each study design in trials 1 and 2 by classification difficulty.

	Trial 1					Trial 2			
	Proportion correctly identified					Proportion correctly identified			
	0.03c	0.05c	0.03c_500_90%		0.03c_5000_99%	0.03c	0.05c	0.03c_500_90%	0.03c_5000_99%
**Mildly abnormal (N = 1200)**	57%	64%	55%		72%	67%	69%	67%	79%
**Normal (N = 600)**	77%	75%	87%		64%	87%	86%	89%	52%
**Severely abnormal (N = 200)**	96%	92%	90%		99%	99%	96%	98%	99%
	**Specificity**					**Specificity**			
	**0.03c**	**0.05c**	**0.03c_500_90%**	**0.03c_5000_99%**		**0.03c**	**0.05c**	**0.03c_500_90%**	**0.03c_5000_99%**
**Normal-Mildly abnormal (Difficult classification)**	74%	68%	85%	64%		87%	86%	89%	52%
**Normal- Severely abnormal (Easy classification)**	74%	68%	85%	64%		87%	86%	89%	52%
**Normal-Abnormal (All)**	74%	68%	85%	64%		87%	86%	89%	52%
	**Sensitivity**					**Sensitivity**			
	**0.03c**	**0.05c**	**0.03c_500_90%**	**0.03c_5000_99%**		**0.03c**	**0.05c**	**0.03c_500_90%**	**0.03c_5000_99%**
**Normal-Mildly abnormal (Difficult classification)**	61%	70%	61%	72%		67%	69%	67%	79%
**Normal- Severely abnormal (Easy classification)**	98%	99%	98%	99%		99%	96%	98%	98%
**Normal-Abnormal (All)**	66%	74%	66%	76%		72%	73%	72%	82%
	**Area under the ROC curve (AUC)**					**Area under the ROC curve (AUC)**			
	**0.03c**	**0.05c**	**0.03c_500_90%**		**0.03c_5000_99%**	**0.03c**	**0.05c**	**0.03c_500_90%**	**0.03c_5000_99%**
**AUC (95%CI): Normal-Mildly abnormal (Difficult classification)**	0.678(0.656–0.700 )	0.692(0.669–0.715 )	0.731(0.711–0.751 )		0.681(0.658–0.704 )	0.771(0.752–0.790)	0.777(0.758–0.796)	0.784(0.766–0.802)	0.656(0.634–0.680)
**AUC (95%CI): Normal-Severely abnormal (Easy classification)**	0.871(0.850–0.889)	0.833(0.813–0.854 )	0.915(0.895–0.930)		0.819(0.799–0.839 )	0.929(0.913–0.944)	0.91(0.891–0.929)	0.938(0.922–0.953)	0.754(0.732–0.776)
**AUC (95%CI): Normal-Abnormal (All)**	0.704(0.683–0.724)	0.712(0.692–0.732)	0.757(0.738–0.776)		0.701(0.680–0.721)	0.794(0.776–0.811)	0.796(0.778–0.814)	0.806(0.789–0.823)	0.671(0.648–0.693)

(0.03c = study design 1; 0.05c = study design 2; 0.03c_500_90% = study design 3; 0.03c_5000_99% = study design 4).

**Figure 1:**
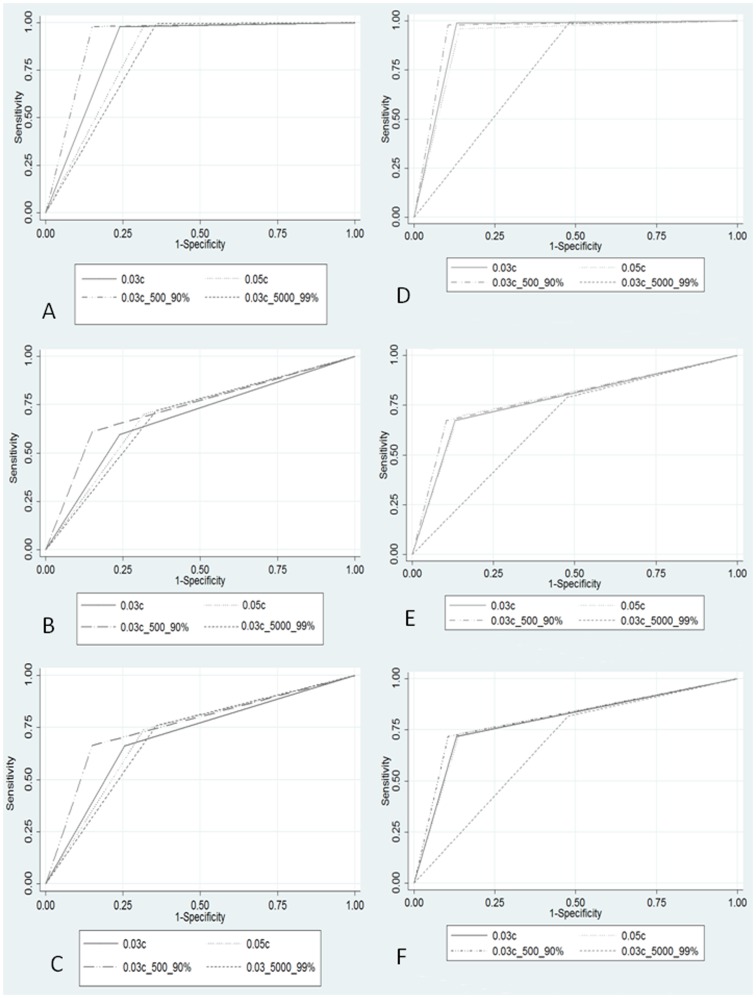
Comparative graphical illustration of the AUC for all classifications by study design (normal-abnormal) - Trial 1 (A) and Trial 2 (D); Comparative graphical illustration of the AUC for easy classifications (normal versus severely abnormal) by study design- Trial 1 (B) and Trial 2 (E); Comparative graphical illustration of the AUC for difficult classifications (normal versus mildly abnormal) by study design- Trial 1 (C) and Trial 2 (F).

The AUC is illustrated in figure 1 both for all study designs and grouped by easy (normal versus severely abnormal) and difficult (normal versus mildly abnormal) classification in both trials. In trial 1, all study designs had an AUC of 0.701 or greater for classification of normal/abnormal. The highest AUC for normal/abnormal classification was 0.757 for those with moderate HIT experience (study design 3). Pairwise comparison between study designs demonstrated a significantly higher AUC for study design 3 compared with each of the other study designs in the classification of normal/abnormal. (p<0.001) This was also demonstrated when comparing the AUC of each study design stratified by easy and difficult classification. (p<0.001) There were no other statistically significant differences in pairwise comparison of study designs. In trial 2, the AUC ranged between 0.671–0.806 for classification of normal/abnormal in all study designs. In both trials, the study design with the lowest AUC was interestingly the design with most experience and highest approval rating (study design 4). This was due to a low true positive rate when classifying normal images (52–64% - Table 2). Pairwise comparison between study designs in trial 2 demonstrated a significantly lower AUC for study design 4 compared with each of the other study designs in the classification of normal/abnormal, as well as easy and difficult classification. (p<0.001) Similar to trial 1, study design 3 demonstrated the highest overall AUC. Paired comparison of study designs between trial 1 and 2 demonstrated a significantly higher AUC for normal-abnormal classification (p<0.001) in trial 2 for all study designs with the exception of study design 4, where trial 1 had a higher AUC (p = 0.004).

Examining the responses from majority of KWs (>50% of KWs) across both trials highlighted that all severely abnormal images were correctly classified. The majority of KWs correctly classified between 64–86% of any abnormal image in trial 1 and between 74–97% in trial 2 (Table 3).

**Table 3:** The percentage of HITs correctly classified by the majority (>50%) of KW’s, with range of percentage of correct “votes” for each image category in brackets.

Trial 1	0.03c	0.05c	0.03c_500_90%	0.03c_5000_99%
Normal (N = 30)	90%(25–95)	87%(30–90)	97%(50–100)	90%(30–90)
Mildly abnormal (N = 60)	58%(25–95)	83%(25–100)	63%(20–100)	80%(35–100)
Severely abnormal (N = 10)	100%(90–100)	100%(90–100)	100%(90–100)	100%(95–100)
Any abnormality (N = 70)	64%(25–100)	86%(25–100)	69%(20–100)	83%(35–100)
**Trial 2**	**0.03c**	**0.05c**	**0.03c_500_90%**	**0.03c_5000_99%**
Normal (N = 30)	97%(50–100)	97%(40–100)	97%(45–100)	50%(30–75)
Mildly abnormal (N = 60)	68%(10–100)	85%(20–100)	70%(15–100)	96%(45–100)
Severely abnormal (N = 10)	100%(95–100)	100%(95–100)	100%(95–100)	100%(95–100)
Any abnormality (N = 70)	80%(10–100)	87%(20–100)	74%(15–100)	97%(45–100)

**Figure 2:**
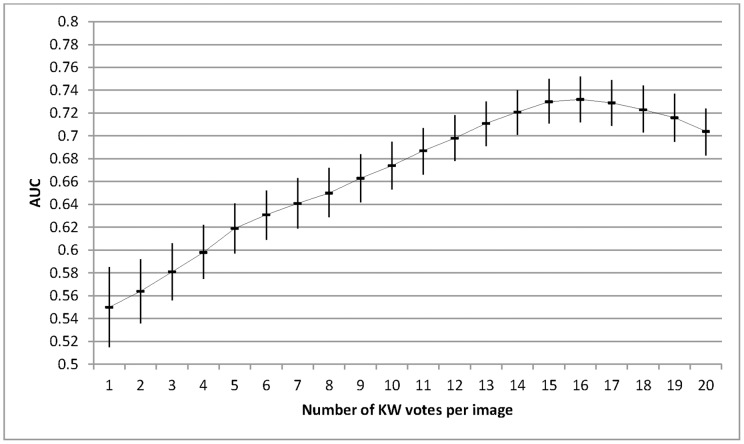
The AUC and associated 95%CI for trial 1 (0.03c) as a function of the number of KW gradings per image. The AUC increases as the number of KW gradings increases with a peak at 16 individual gradings per image. A similar curve was obtained for all study designs in both trials, although a variation was seen in the optimal number of KWs needed to achieve a peak ROC.

The AUC varied depending on the number of individual KW gradings per image. For study design 1 (0.03c) in trial 1, the AUC rose steadily peaking at 16 gradings per image, diminishing slightly thereafter. (Figure 2) The overall relationship between a higher AUC and a larger number of KWs was similar in all study designs and in both trials. However, there was a variation in the optimal number of KWs needed to achieve the highest ROC. For trial 1, this varied between 11–16 KWs and for trial 2 this ranged between 11–20 KWs.

## Discussion

This study demonstrates that crowdsourcing is a potentially effective, viable and inexpensive method for the preliminary analysis of fundus photographs. Identification of severe abnormalities is particularly accurate with a sensitivity of ≥98%, and a high AUC estimate (range 0.819–0.915) which was replicated in the second trial (AUC range: 0.754–0.938). The ability to distinguish between normal and mildly abnormal images had a sensitivity ranging between 61–72% and between 64–86% of any abnormal image was correctly classified by over half of all KWs. In trial 2, these findings were replicated and compared favourably with trial 1.

Several interesting features of distributed human intelligence tasks should be noted. Using an unselected crowdsource, we received 2,000 classifications at a total cost of $60 in under 24 hours, highlighting the power of this technique for rapid cost-effective data analysis. In line with previous reports, increased incentive did not necessarily lead to increased accuracy [9] and increasing the number of KW gradings and the KW experience did not have a simple relationship with classification accuracy.

Population screening for common diseases such as diabetic retinopathy can be costly and time-consuming, with increasing research emphasis being placed on automated or semi-automated grading. [10] Sanchez et al [11] recently compared computer automated detection (CAD) and expert grading for diabetic retinopathy based on non-mydriatic fundus photography. They reported no difference in accuracy between the expert graders and CAD, with an AUC for computer aided detection of 0.721–0.973 depending on the difficulty of classification. Other studies have similarly demonstrated an AUC ranging between 0.812–0.839 for automated detection of early diabetic retinopathy. [12] These recent studies are comparable to our crowdsourced data, with an AUC ranging between 0.731–0.915 for abnormal versus normal depending on classification difficulty. Similarly, our results compare favourably to automated techniques for detection of age-related macular degeneration where a sensitivity of >94% was found for severe disease. [13].

Recent compelling clinical examples of crowdsourcing have also demonstrated a high level of diagnostic accuracy equalling results from expert graders. [4], [6] Expert graders of mild retinopathy of prematurity have recently reported an AUC of 0.84 [14], which is higher than the AUC for mildly abnormal image classification identified in this study (0.656–0.777 across both trials). However, our results compare favourably with other studies, where expert grading of grade 1 diabetic retinopathy demonstrated an AUC range of between 0.623–0.789. [11] Furthermore, an analysis of the automated detection of drusen, cotton-wool spots, exudates and bright retinal lesions reported an expert grading sensitivity of between 87–95% depending on the type of lesion [15], which is higher than our finding for mildly abnormal detection (61–79%), but lower than the range for severely abnormal detection (96–99%). Findings from this study should be interpreted with certain considerations. By design, our data was heavily biased towards mildly abnormal images which comprised 60 out of 100 images. Distinguishing normal from mildly abnormal is the most difficult classification task. In addition, our instructions were kept simple, with very limited examples and training provided to the crowdsourced participants. Additional training exercises and examples are likely improve the classification accuracy. All KWs meeting the eligibility requirements were allowed to participate in the trials, thus a proportion of individuals may have participated in both trials, however based on the rapidity of the response and the low mean number of HITs performed by each KW we expect this number to be low. Moreover, it should be noted that there are limitations to using crowdsourcing as a tool. The individuals classifying the images are unknown and may represent a stratified subgroup with a risk of inherent bias, [9] and ethical issues surrounding the release and online access of anonymised clinical data can be complex. The availability of robust anonymisation tools for the analysis of large clinical datasets may facilitate the uptake of crowdsourcing methods and ensure compliance with patient confidentiality. [16].

Nonetheless, micro-task markets offer a potential paradigm for engaging a large number of users for low time and monetary costs. With minimal training, crowdsourcing represents an effective, repeatable, rapid and cost-effective method of retinal image analysis. Based on our study, the accuracy obtained from crowdsourcing retinal image analysis compared to a gold standard is at least comparable both to computer automated techniques in disease detection and some reports from expert graders. Further work is needed to compare the diagnostic accuracy of specific disease detection tasks between a crowdsource and expert graders. The ideal crowdsource remuneration and categorization skill required remains uncertain, however in this task, a moderate skill level provided a higher accuracy than both unskilled and highly skilled KWs in the detection of mild and severe disease. Larger studies with more comprehensive crowdsource training are needed to explore the utility of this novel technique in large scale medical image analysis.

## Supporting Information

Figure S1 **An example of a typical questionnaire each KW was asked to complete.**
(DOC)

Figure S2 **An example of an image with a mild abnormality.**
(DOC)

Figure S3 **An example of an image with a mild abnormality.**
(DOC)

Figure S4 **An example of an image with a severe abnormality.**
(DOC)
