# Supplementary figures and images for: Crowdsourcing as a Novel Technique for Retinal Fundus Photography Classification: Analysis of Images in the EPIC Norfolk Cohort on Behalf of the UKBiobank Eye and Vision Consortium
Source: PLoS One. 2013 Aug 21;8(8):e71154. doi: 10.1371/journal.pone.0071154 (PMC3749186; doi:10.1371/journal.pone.0071154)

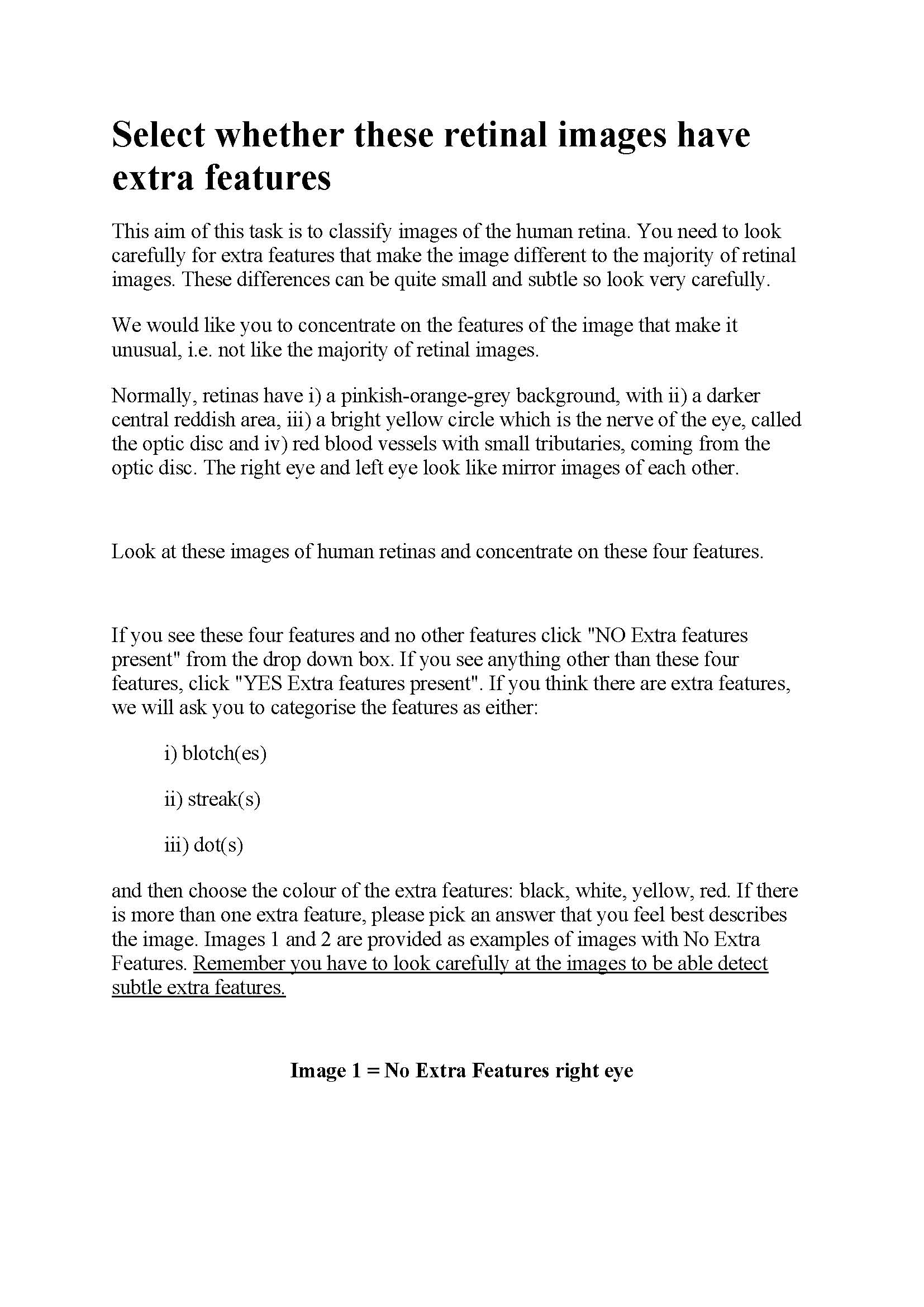


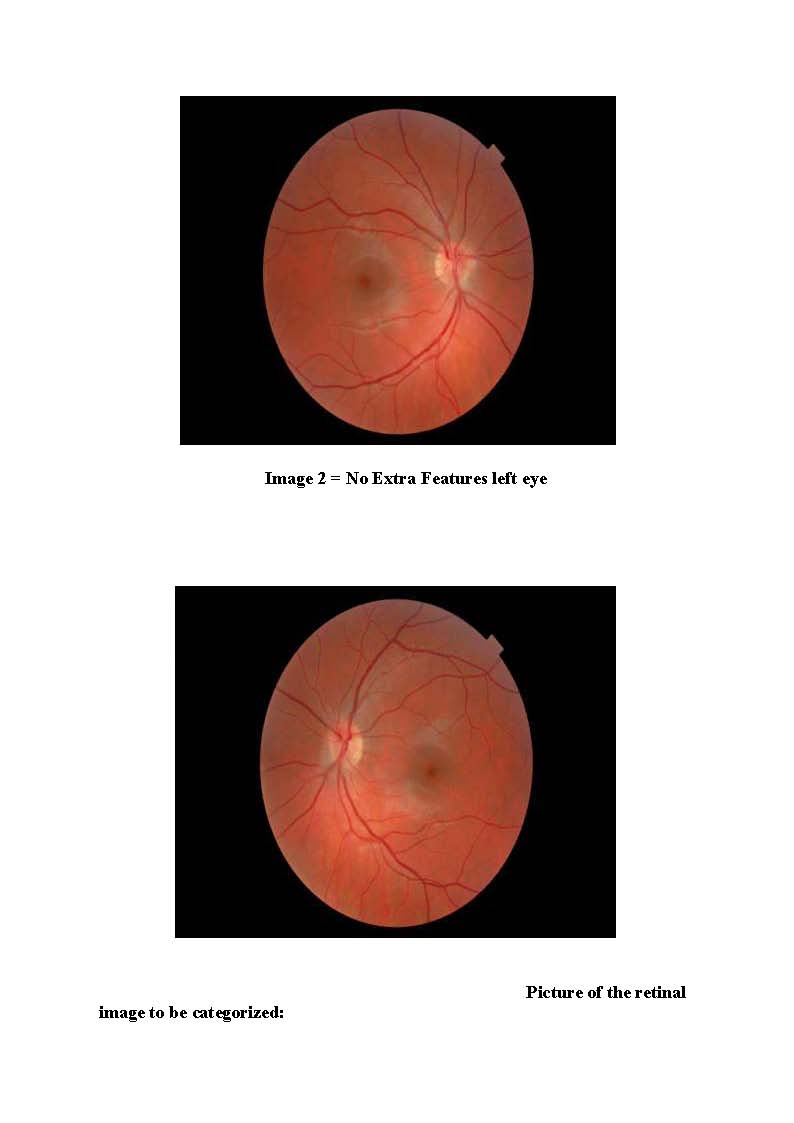

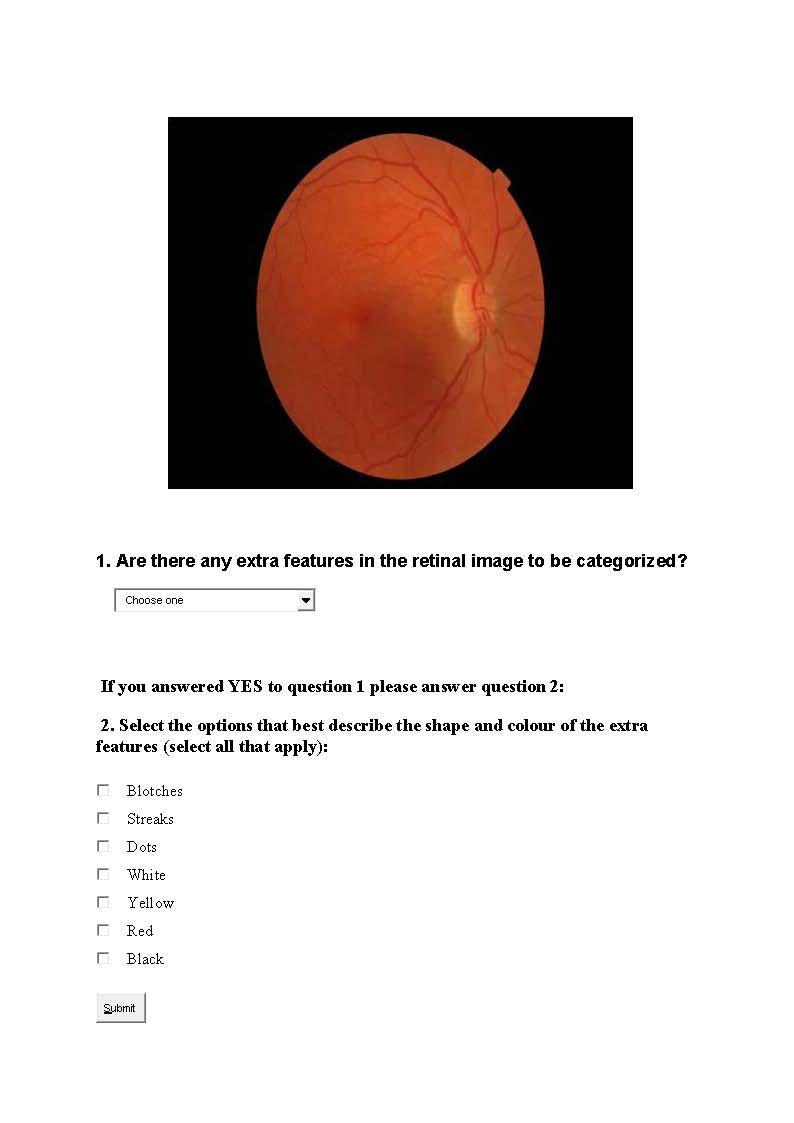


Figure S1 – An example of a typical questionnaire each KW was asked to complete.

Supplement: Figure S1 — An example of a typical questionnaire each KW was asked to complete. (DOC) [file pone.0071154.s001.doc]

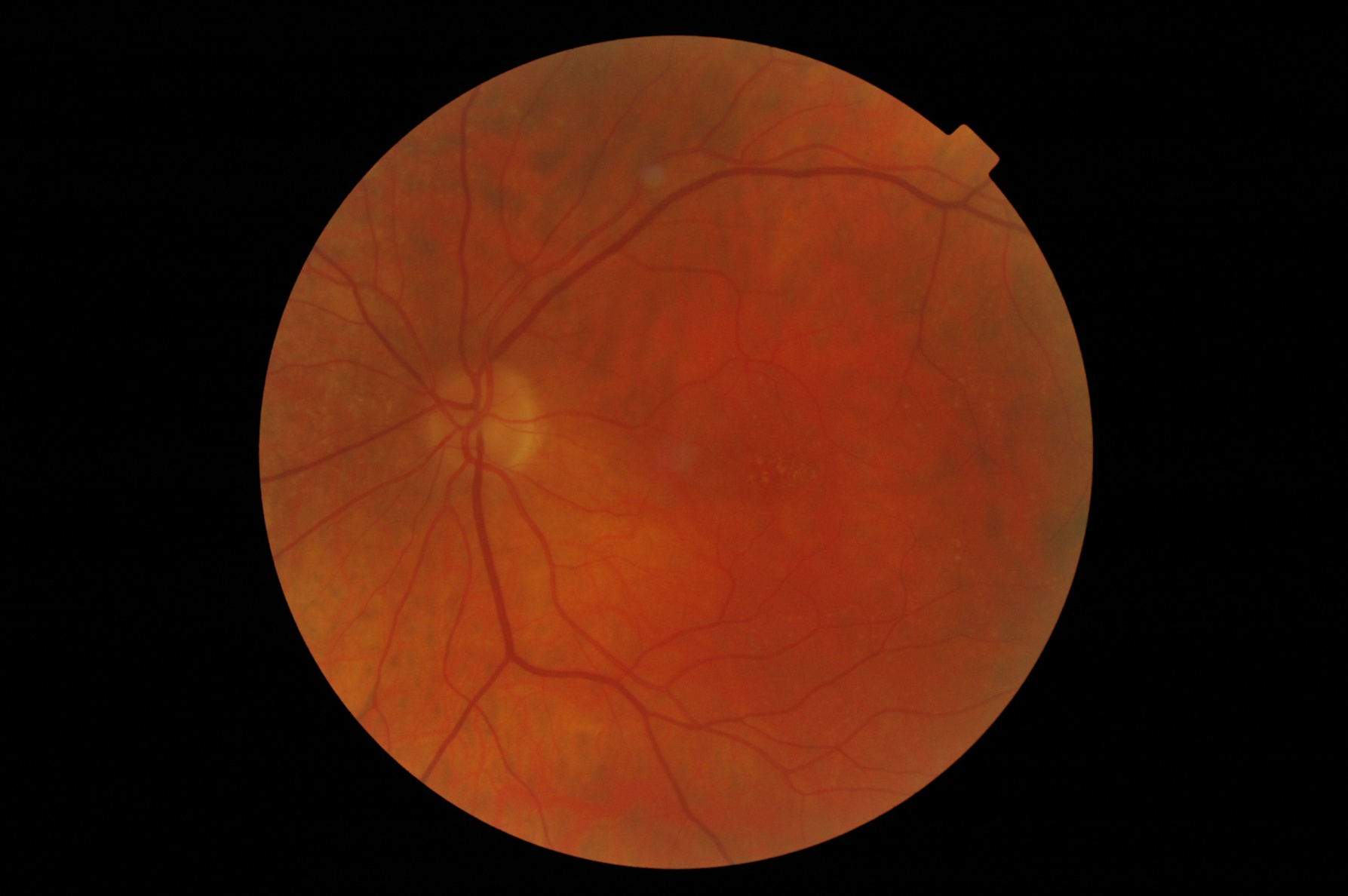


Figure S2 – An example of an image with a mild abnormality

Supplement: Figure S2 — An example of an image with a mild abnormality. (DOC) [file pone.0071154.s002.doc]

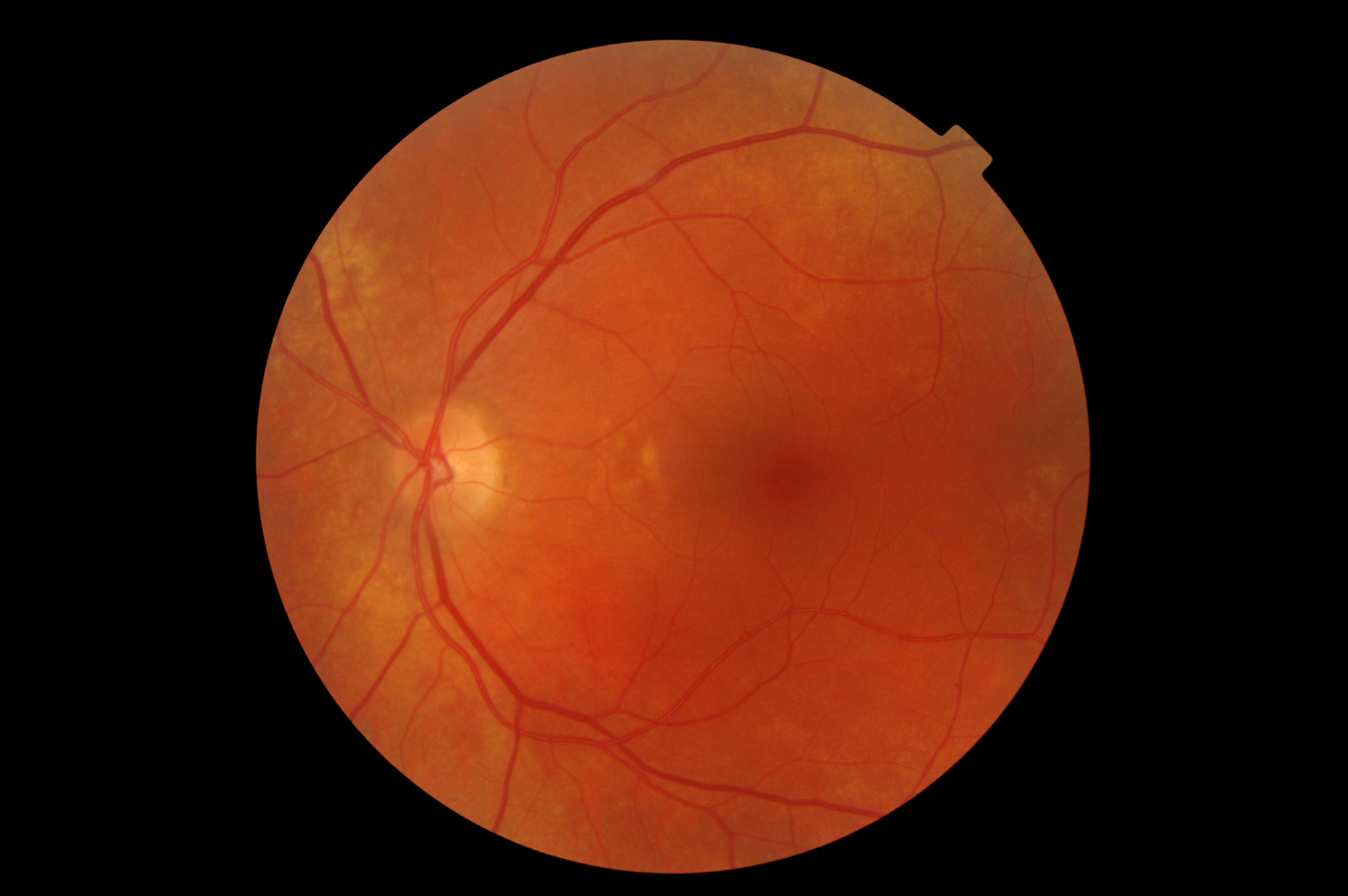


Figure S3 – An example of an image with a mild abnormality

Supplement: Figure S3 — An example of an image with a mild abnormality. (DOC) [file pone.0071154.s003.doc]

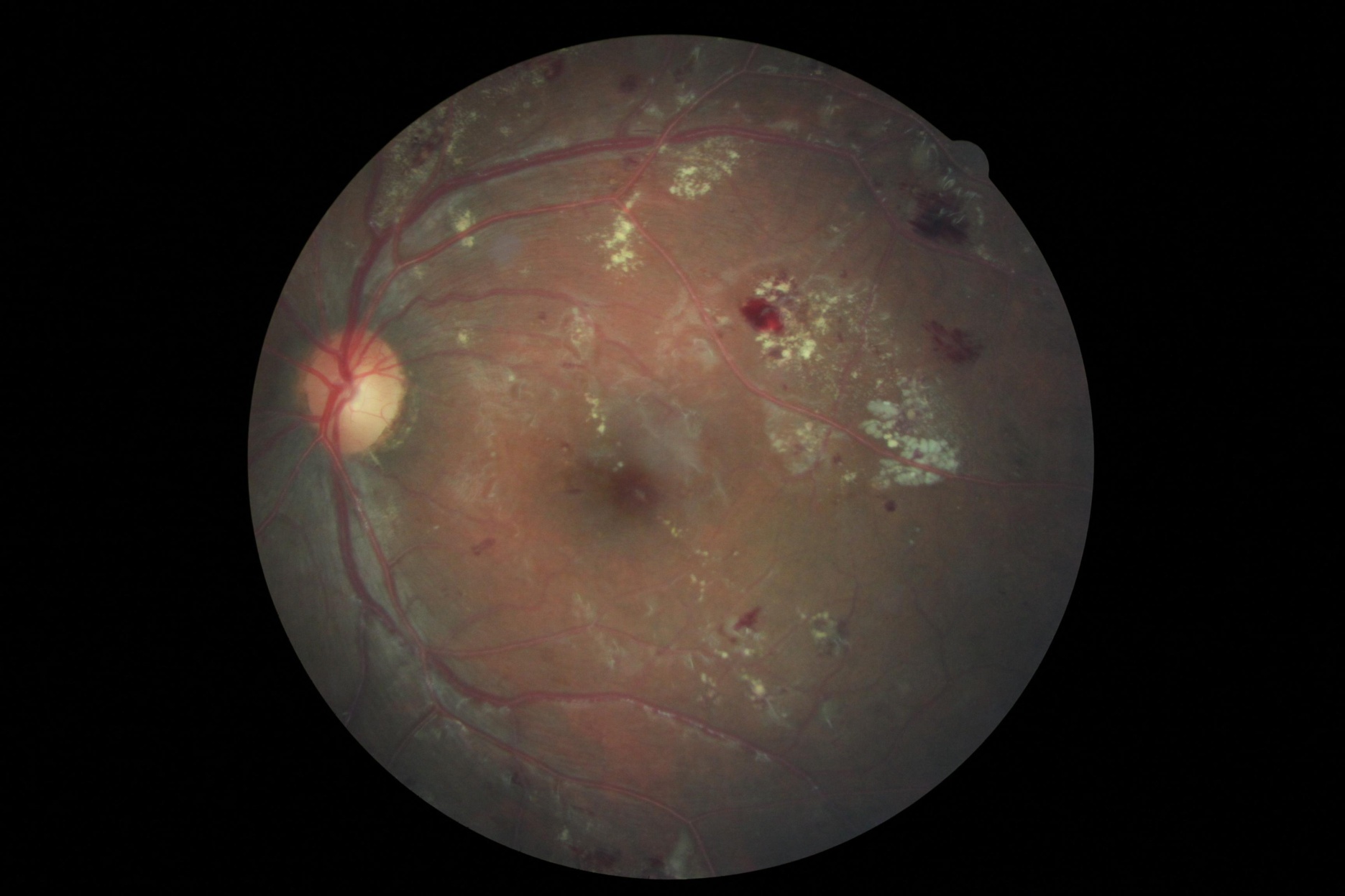


Figure S4 – An example of an image with a severe abnormality

Supplement: Figure S4 — An example of an image with a severe abnormality. (DOC) [file pone.0071154.s004.doc]
